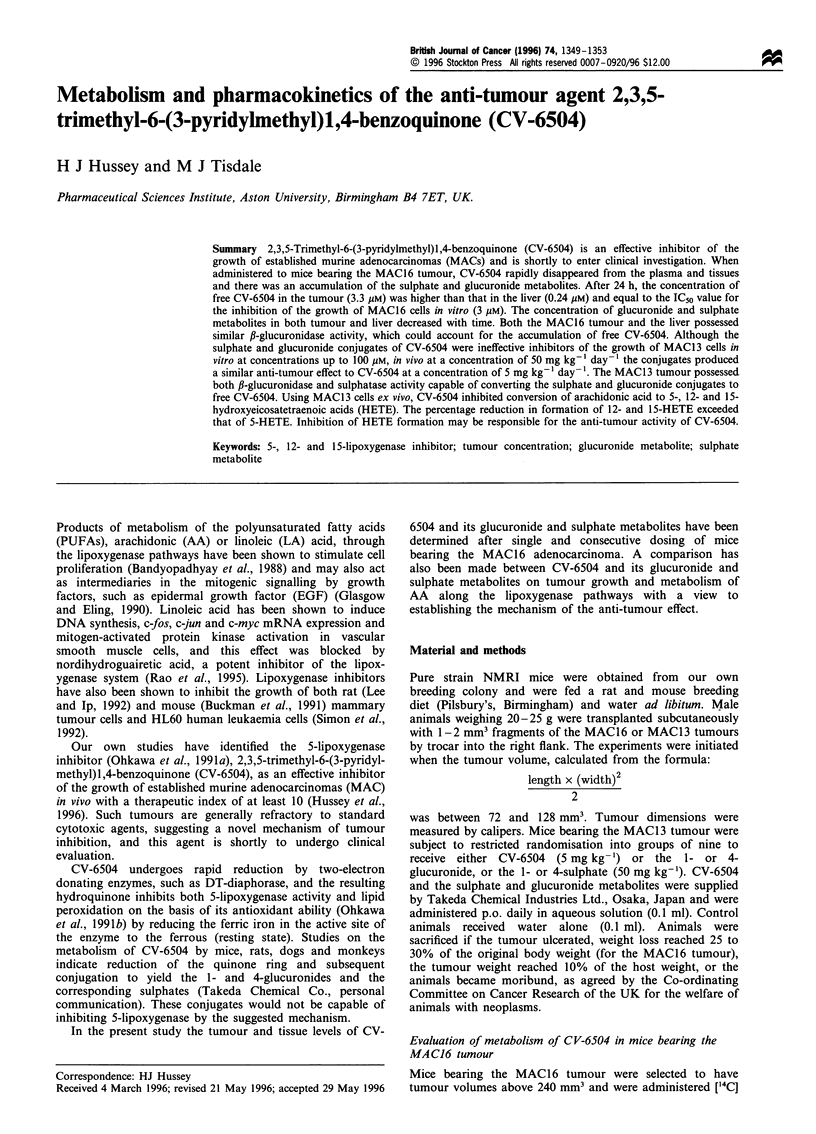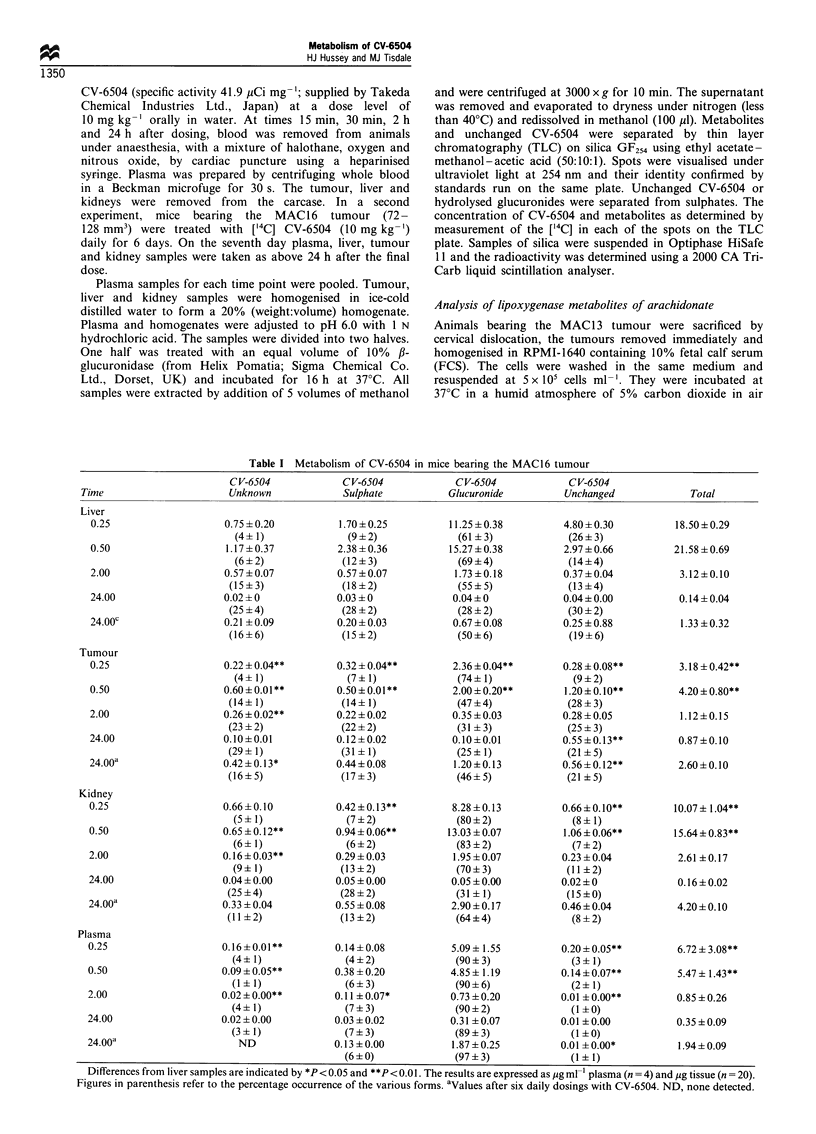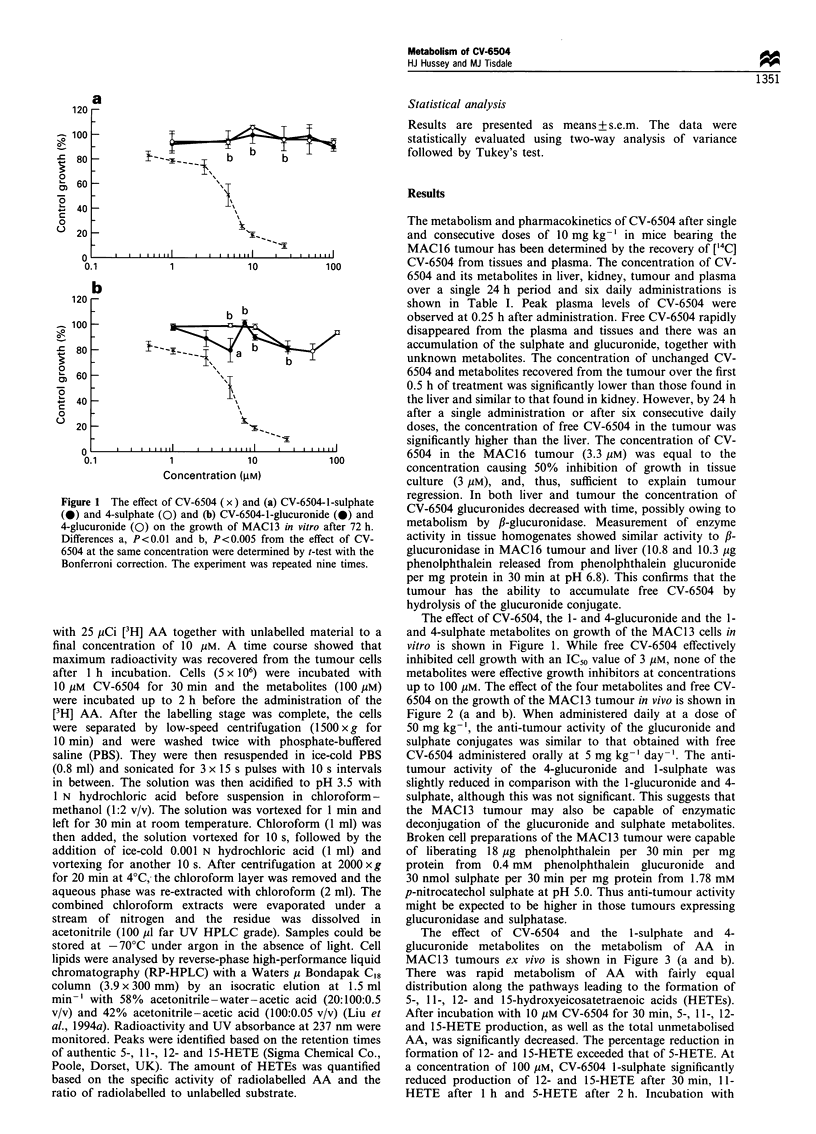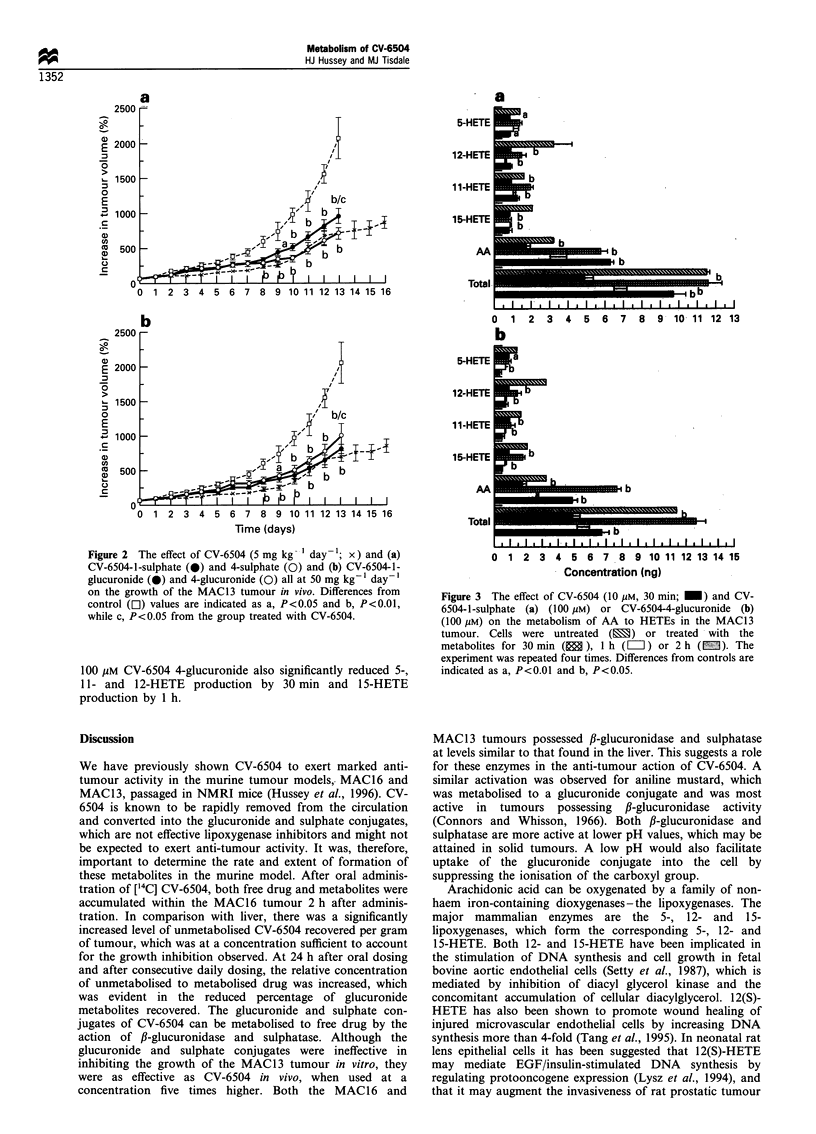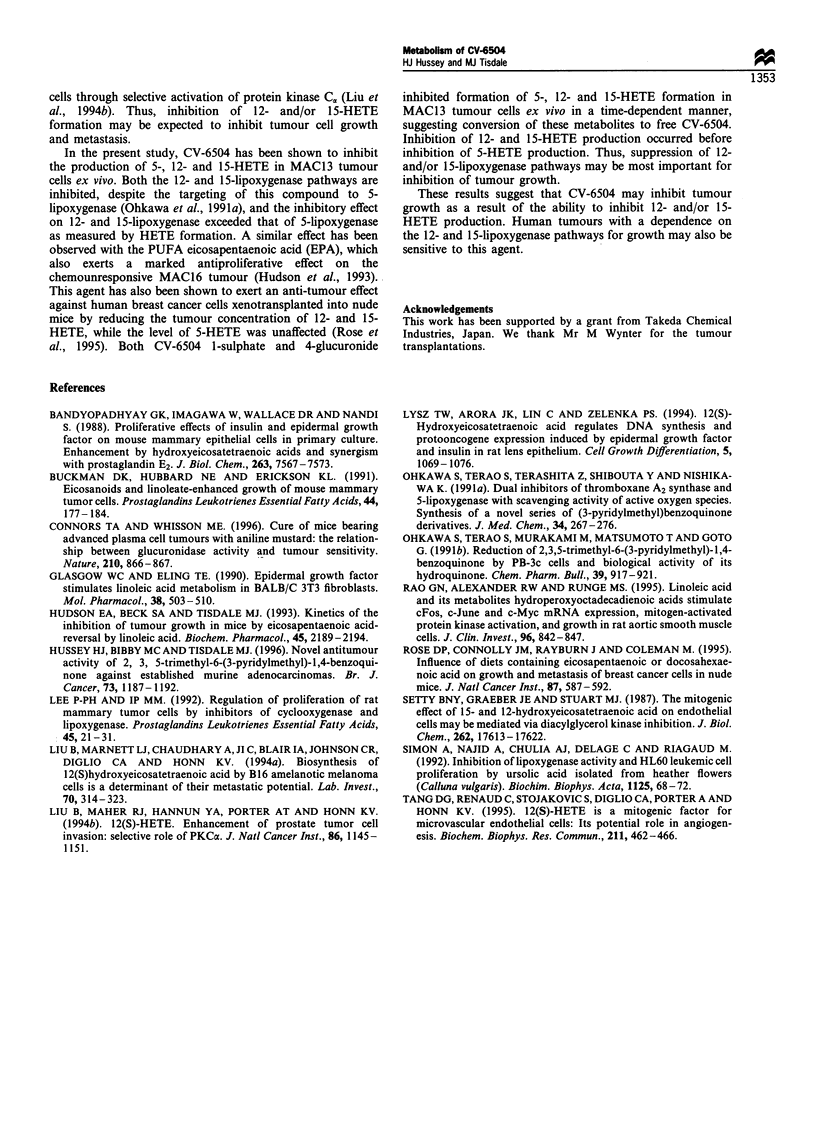# Metabolism and pharmacokinetics of the anti-tumour agent 2,3,5-trimethyl-6-(3-pyridylmethyl)1,4-benzoquinone (CV-6504).

**DOI:** 10.1038/bjc.1996.548

**Published:** 1996-11

**Authors:** H. J. Hussey, M. J. Tisdale

**Affiliations:** Pharmaceutical Sciences Institute, Aston University, Birmingham, UK.

## Abstract

2,3,5-Trimethyl-6-(3-pyridylmethyl)1,4-benzoquinone (CV-6504) is an effective inhibitor of the growth of established murine adenocarcinomas (MACs) and is shortly to enter clinical investigation. When administered to mice bearing the MAC16 tumour, CV-6504 rapidly disappeared from the plasma and tissues and there was an accumulation of the sulphate and glucuronide metabolites. After 24 h, the concentration of free CV-6504 in the tumour (3.3 microM) was higher than that in the liver (0.24 microM) and equal to the IC50 value for the inhibition of the growth of MAC16 cells in vitro (3 microM). The concentration of glucuronide and sulphate metabolites in both tumour and liver decreased with time. Both the MAC16 tumour and the liver possessed similar beta-glucuronidase activity, which could account for the accumulation of free CV-6504. Although the sulphate and glucuronide conjugates of CV-6504 were ineffective inhibitors of the growth of MAC13 cells in vitro at concentrations up to 100 microM, in vivo at a concentration of 50 mg kg-1 day-1 the conjugates produced a similar anti-tumour effect to CV-6504 at a concentration of 5 mg kg-1 day-1. The MAC13 tumour possessed both beta-glucuronidase and sulphatase activity capable of converting the sulphate and glucuronide conjugates to free CV-6504. Using MAC13 cells ex vivo, CV-6504 inhibited conversion of arachidonic acid to 5-, 12- and 15-hydroxyeicosatetraenoic acids (HETE). The percentage reduction in formation of 12- and 15-HETE exceeded that of 5-HETE. Inhibition of HETE formation may be responsible for the anti-tumour activity of CV-6504.


British Journal of Cancer (1996) 74, 1349–1353
© 1996 Stockton Press All rights reserved 0007–0920/96 $12.00

### Metabolism and pharmacokinetics of the anti-tumour agent 2,3,5-trimethyl-6-(3-pyridylmethyl)1,4-benzoquinone (CV-6504)

H J Hussey and M J Tisdale

*Pharmaceutical Sciences Institute, Aston University, Birmingham B4 7ET, UK.*

**Summary** 2,3,5-Trimethyl-6-(3-pyridylmethyl)1,4-benzoquinone (CV-6504) is an effective inhibitor of the growth of established murine adenocarcinomas (MACs) and is shortly to enter clinical investigation. When administered to mice bearing the MAC16 tumour, CV-6504 rapidly disappeared from the plasma and tissues and there was an accumulation of the sulphate and glucuronide metabolites. After 24 h, the concentration of free CV-6504 in the tumour (3.3 $\mu$M) was higher than that in the liver (0.24 $\mu$M) and equal to the $IC_{50}$ value for the inhibition of the growth of MAC16 cells *in vitro* (3 $\mu$M). The concentration of glucuronide and sulphate metabolites in both tumour and liver decreased with time. Both the MAC16 tumour and the liver possessed similar $\beta$-glucuronidase activity, which could account for the accumulation of free CV-6504. Although the sulphate and glucuronide conjugates of CV-6504 were ineffective inhibitors of the growth of MAC13 cells *in vitro* at concentrations up to 100 $\mu$M, *in vivo* at a concentration of 50 mg $kg^{-1}$ $day^{-1}$ the conjugates produced a similar anti-tumour effect to CV-6504 at a concentration of 5 mg $kg^{-1}$ $day^{-1}$. The MAC13 tumour possessed both $\beta$-glucuronidase and sulphatase activity capable of converting the sulphate and glucuronide conjugates to free CV-6504. Using MAC13 cells *ex vivo*, CV-6504 inhibited conversion of arachidonic acid to 5-, 12- and 15-hydroxyeicosatetraenoic acids (HETE). The percentage reduction in formation of 12- and 15-HETE exceeded that of 5-HETE. Inhibition of HETE formation may be responsible for the anti-tumour activity of CV-6504.

**Keywords:** 5-, 12- and 15-lipoxygenase inhibitor; tumour concentration; glucuronide metabolite; sulphate metabolite

Products of metabolism of the polyunsaturated fatty acids (PUFAs), arachidonic (AA) or linoleic (LA) acid, through the lipoxygenase pathways have been shown to stimulate cell proliferation (Bandyopadhyay *et al.*, 1988) and may also act as intermediaries in the mitogenic signalling by growth factors, such as epidermal growth factor (EGF) (Glasgow and Eling, 1990). Linoleic acid has been shown to induce DNA synthesis, c-*fos*, c-*jun* and c-*myc* mRNA expression and mitogen-activated protein kinase activation in vascular smooth muscle cells, and this effect was blocked by nordihydroguairetic acid, a potent inhibitor of the lipoxygenase system (Rao *et al.*, 1995). Lipoxygenase inhibitors have also been shown to inhibit the growth of both rat (Lee and Ip, 1992) and mouse (Buckman *et al.*, 1991) mammary tumour cells and HL60 human leukaemia cells (Simon *et al.*, 1992).

Our own studies have identified the 5-lipoxygenase inhibitor (Ohkawa *et al.*, 1991*a*), 2,3,5-trimethyl-6-(3-pyridylmethyl)1,4-benzoquinone (CV-6504), as an effective inhibitor of the growth of established murine adenocarcinomas (MAC) *in vivo* with a therapeutic index of at least 10 (Hussey *et al.*, 1996). Such tumours are generally refractory to standard cytotoxic agents, suggesting a novel mechanism of tumour inhibition, and this agent is shortly to undergo clinical evaluation.

CV-6504 undergoes rapid reduction by two-electron donating enzymes, such as DT-diaphorase, and the resulting hydroquinone inhibits both 5-lipoxygenase activity and lipid peroxidation on the basis of its antioxidant ability (Ohkawa *et al.*, 1991*b*) by reducing the ferric iron in the active site of the enzyme to the ferrous (resting state). Studies on the metabolism of CV-6504 by mice, rats, dogs and monkeys indicate reduction of the quinone ring and subsequent conjugation to yield the 1- and 4-glucuronides and the corresponding sulphates (Takeda Chemical Co., personal communication). These conjugates would not be capable of inhibiting 5-lipoxygenase by the suggested mechanism.

In the present study the tumour and tissue levels of CV-6504 and its glucuronide and sulphate metabolites have been determined after single and consecutive dosing of mice bearing the MAC16 adenocarcinoma. A comparison has also been made between CV-6504 and its glucuronide and sulphate metabolites on tumour growth and metabolism of AA along the lipoxygenase pathways with a view to establishing the mechanism of the anti-tumour effect.

### Material and methods

Pure strain NMRI mice were obtained from our own breeding colony and were fed a rat and mouse breeding diet (Pilsbury's, Birmingham) and water *ad libitum.* Male animals weighing 20–25 g were transplanted subcutaneously with 1–2 $mm^3$ fragments of the MAC16 or MAC13 tumours by trocar into the right flank. The experiments were initiated when the tumour volume, calculated from the formula:

$$\frac{\text{length} \times (\text{width})^2}{2}$$

was between 72 and 128 $mm^3$. Tumour dimensions were measured by calipers. Mice bearing the MAC13 tumour were subject to restricted randomisation into groups of nine to receive either CV-6504 (5 mg $kg^{-1}$) or the 1- or 4-glucuronide, or the 1- or 4-sulphate (50 mg $kg^{-1}$). CV-6504 and the sulphate and glucuronide metabolites were supplied by Takeda Chemical Industries Ltd., Osaka, Japan and were administered p.o. daily in aqueous solution (0.1 ml). Control animals received water alone (0.1 ml). Animals were sacrificed if the tumour ulcerated, weight loss reached 25 to 30% of the original body weight (for the MAC16 tumour), the tumour weight reached 10% of the host weight, or the animals became moribund, as agreed by the Co-ordinating Committee on Cancer Research of the UK for the welfare of animals with neoplasms.

### *Evaluation of metabolism of CV-6504 in mice bearing the MAC16 tumour*

Mice bearing the MAC16 tumour were selected to have tumour volumes above 240 $mm^3$ and were administered [$^{14}$C]

Correspondence: HJ Hussey
Received 4 March 1996; revised 21 May 1996; accepted 29 May 1996

CV-6504 (specific activity 41.9 μCi mg$^{-1}$; supplied by Takeda Chemical Industries Ltd., Japan) at a dose level of 10 mg kg$^{-1}$ orally in water. At times 15 min, 30 min, 2 h and 24 h after dosing, blood was removed from animals under anaesthesia, with a mixture of halothane, oxygen and nitrous oxide, by cardiac puncture using a heparinised syringe. Plasma was prepared by centrifuging whole blood in a Beckman microfuge for 30 s. The tumour, liver and kidneys were removed from the carcase. In a second experiment, mice bearing the MAC16 tumour (72–128 mm$^3$) were treated with [$^{14}$C] CV-6504 (10 mg kg$^{-1}$) daily for 6 days. On the seventh day plasma, liver, tumour and kidney samples were taken as above 24 h after the final dose.

Plasma samples for each time point were pooled. Tumour, liver and kidney samples were homogenised in ice-cold distilled water to form a 20% (weight:volume) homogenate. Plasma and homogenates were adjusted to pH 6.0 with 1 N hydrochloric acid. The samples were divided into two halves. One half was treated with an equal volume of 10% β-glucuronidase (from Helix Pomatia; Sigma Chemical Co. Ltd., Dorset, UK) and incubated for 16 h at 37°C. All samples were extracted by addition of 5 volumes of methanol and were centrifuged at 3000 × *g* for 10 min. The supernatant was removed and evaporated to dryness under nitrogen (less than 40°C) and redissolved in methanol (100 μl). Metabolites and unchanged CV-6504 were separated by thin layer chromatography (TLC) on silica $GF_{254}$ using ethyl acetate–methanol–acetic acid (50:10:1). Spots were visualised under ultraviolet light at 254 nm and their identity confirmed by standards run on the same plate. Unchanged CV-6504 or hydrolysed glucuronides were separated from sulphates. The concentration of CV-6504 and metabolites as determined by measurement of the [$^{14}$C] in each of the spots on the TLC plate. Samples of silica were suspended in Optiphase HiSafe 11 and the radioactivity was determined using a 2000 CA Tri-Carb liquid scintillation analyser.

### *Analysis of lipoxygenase metabolites of arachidonate*

Animals bearing the MAC13 tumour were sacrificed by cervical dislocation, the tumours removed immediately and homogenised in RPMI-1640 containing 10% fetal calf serum (FCS). The cells were washed in the same medium and resuspended at 5 × 10$^5$ cells ml$^{-1}$. They were incubated at 37°C in a humid atmosphere of 5% carbon dioxide in air

**Table I** Metabolism of CV-6504 in mice bearing the MAC16 tumour

| *Time* | *CV-6504 Unknown* | *CV-6504 Sulphate* | *CV-6504 Glucuronide* | *CV-6504 Unchanged* | *Total* |
|---|---|---|---|---|---|
| Liver | | | | | |
| 0.25 | 0.75 ± 0.20 (4 ± 1) | 1.70 ± 0.25 (9 ± 2) | 11.25 ± 0.38 (61 ± 3) | 4.80 ± 0.30 (26 ± 3) | 18.50 ± 0.29 |
| 0.50 | 1.17 ± 0.37 (6 ± 2) | 2.38 ± 0.36 (12 ± 3) | 15.27 ± 0.38 (69 ± 4) | 2.97 ± 0.66 (14 ± 4) | 21.58 ± 0.69 |
| 2.00 | 0.57 ± 0.07 (15 ± 3) | 0.57 ± 0.07 (18 ± 2) | 1.73 ± 0.18 (55 ± 5) | 0.37 ± 0.04 (13 ± 4) | 3.12 ± 0.10 |
| 24.00 | 0.02 ± 0 (25 ± 4) | 0.03 ± 0 (28 ± 2) | 0.04 ± 0 (28 ± 2) | 0.04 ± 0.00 (30 ± 2) | 0.14 ± 0.04 |
| 24.00[c] | 0.21 ± 0.09 (16 ± 6) | 0.20 ± 0.03 (15 ± 2) | 0.67 ± 0.08 (50 ± 6) | 0.25 ± 0.88 (19 ± 6) | 1.33 ± 0.32 |
| Tumour | | | | | |
| 0.25 | 0.22 ± 0.04** (4 ± 1) | 0.32 ± 0.04** (7 ± 1) | 2.36 ± 0.04** (74 ± 1) | 0.28 ± 0.08** (9 ± 2) | 3.18 ± 0.42** |
| 0.50 | 0.60 ± 0.01** (14 ± 1) | 0.50 ± 0.01** (14 ± 1) | 2.00 ± 0.20** (47 ± 4) | 1.20 ± 0.10** (28 ± 3) | 4.20 ± 0.80** |
| 2.00 | 0.26 ± 0.02** (23 ± 2) | 0.22 ± 0.02 (22 ± 2) | 0.35 ± 0.03 (31 ± 3) | 0.28 ± 0.05 (25 ± 3) | 1.12 ± 0.15 |
| 24.00 | 0.10 ± 0.01 (29 ± 1) | 0.12 ± 0.02 (31 ± 1) | 0.10 ± 0.01 (25 ± 1) | 0.55 ± 0.13** (21 ± 5) | 0.87 ± 0.10 |
| 24.00[a] | 0.42 ± 0.13* (16 ± 5) | 0.44 ± 0.08 (17 ± 3) | 1.20 ± 0.13 (46 ± 5) | 0.56 ± 0.12** (21 ± 5) | 2.60 ± 0.10 |
| Kidney | | | | | |
| 0.25 | 0.66 ± 0.10 (5 ± 1) | 0.42 ± 0.13** (7 ± 2) | 8.28 ± 0.13 (80 ± 2) | 0.66 ± 0.10** (8 ± 1) | 10.07 ± 1.04** |
| 0.50 | 0.65 ± 0.12** (6 ± 1) | 0.94 ± 0.06** (6 ± 2) | 13.03 ± 0.07 (83 ± 2) | 1.06 ± 0.06** (7 ± 2) | 15.64 ± 0.83** |
| 2.00 | 0.16 ± 0.03** (9 ± 1) | 0.29 ± 0.03 (13 ± 2) | 1.95 ± 0.07 (70 ± 3) | 0.23 ± 0.04 (11 ± 2) | 2.61 ± 0.17 |
| 24.00 | 0.04 ± 0.00 (25 ± 4) | 0.05 ± 0.00 (28 ± 2) | 0.05 ± 0.00 (31 ± 1) | 0.02 ± 0 (15 ± 0) | 0.16 ± 0.02 |
| 24.00[a] | 0.33 ± 0.04 (11 ± 2) | 0.55 ± 0.08 (13 ± 2) | 2.90 ± 0.17 (64 ± 4) | 0.46 ± 0.04 (8 ± 2) | 4.20 ± 0.10 |
| Plasma | | | | | |
| 0.25 | 0.16 ± 0.01** (4 ± 1) | 0.14 ± 0.08 (4 ± 2) | 5.09 ± 1.55 (90 ± 3) | 0.20 ± 0.05** (3 ± 1) | 6.72 ± 3.08** |
| 0.50 | 0.09 ± 0.05** (1 ± 1) | 0.38 ± 0.20 (6 ± 3) | 4.85 ± 1.19 (90 ± 6) | 0.14 ± 0.07** (2 ± 1) | 5.47 ± 1.43** |
| 2.00 | 0.02 ± 0.00** (4 ± 1) | 0.11 ± 0.07* (7 ± 3) | 0.73 ± 0.20 (90 ± 2) | 0.01 ± 0.00** (1 ± 0) | 0.85 ± 0.26 |
| 24.00 | 0.02 ± 0.00 (3 ± 1) | 0.03 ± 0.02 (7 ± 3) | 0.31 ± 0.07 (89 ± 3) | 0.01 ± 0.00 (1 ± 0) | 0.35 ± 0.09 |
| 24.00[a] | ND | 0.13 ± 0.00 (6 ± 0) | 1.87 ± 0.25 (97 ± 3) | 0.01 ± 0.00* (1 ± 1) | 1.94 ± 0.09 |

Differences from liver samples are indicated by *$P$ < 0.05 and **$P$ < 0.01. The results are expressed as μg ml$^{-1}$ plasma ($n$ = 4) and μg tissue ($n$ = 20). Figures in parenthesis refer to the percentage occurrence of the various forms. [a]Values after six daily dosings with CV-6504. ND, none detected.

**Figure 1** The effect of CV-6504 (×) and (**a**) CV-6504-1-sulphate (●) and 4-sulphate (○) and (**b**) CV-6504-1-glucuronide (●) and 4-glucuronide (○) on the growth of MAC13 *in vitro* after 72 h. Differences a, $P<0.01$ and b, $P<0.005$ from the effect of CV-6504 at the same concentration were determined by *t*-test with the Bonferroni correction. The experiment was repeated nine times.

with 25 μCi [$^3$H] AA together with unlabelled material to a final concentration of 10 μM. A time course showed that maximum radioactivity was recovered from the tumour cells after 1 h incubation. Cells ($5\times10^6$) were incubated with 10 μM CV-6504 for 30 min and the metabolites (100 μM) were incubated up to 2 h before the administration of the [$^3$H] AA. After the labelling stage was complete, the cells were separated by low-speed centrifugation ($1500\times g$ for 10 min) and were washed twice with phosphate-buffered saline (PBS). They were then resuspended in ice-cold PBS (0.8 ml) and sonicated for $3\times15$ s pulses with 10 s intervals in between. The solution was then acidified to pH 3.5 with 1 N hydrochloric acid before suspension in chloroform–methanol (1:2 v/v). The solution was vortexed for 1 min and left for 30 min at room temperature. Chloroform (1 ml) was then added, the solution vortexed for 10 s, followed by the addition of ice-cold 0.001 N hydrochloric acid (1 ml) and vortexing for another 10 s. After centrifugation at $2000\times g$ for 20 min at 4°C, the chloroform layer was removed and the aqueous phase was re-extracted with chloroform (2 ml). The combined chloroform extracts were evaporated under a stream of nitrogen and the residue was dissolved in acetonitrile (100 μl far UV HPLC grade). Samples could be stored at −70°C under argon in the absence of light. Cell lipids were analysed by reverse-phase high-performance liquid chromatography (RP-HPLC) with a Waters μ Bondapak $C_{18}$ column ($3.9\times300$ mm) by an isocratic elution at 1.5 ml min$^{-1}$ with 58% acetonitrile–water–acetic acid (20:100:0.5 v/v) and 42% acetonitrile–acetic acid (100:0.05 v/v) (Liu *et al.*, 1994*a*). Radioactivity and UV absorbance at 237 nm were monitored. Peaks were identified based on the retention times of authentic 5-, 11-, 12- and 15-HETE (Sigma Chemical Co., Poole, Dorset, UK). The amount of HETEs was quantified based on the specific activity of radiolabelled AA and the ratio of radiolabelled to unlabelled substrate.

*Statistical analysis*

Results are presented as means±s.e.m. The data were statistically evaluated using two-way analysis of variance followed by Tukey's test.

### Results

The metabolism and pharmacokinetics of CV-6504 after single and consecutive doses of 10 mg kg$^{-1}$ in mice bearing the MAC16 tumour has been determined by the recovery of [$^{14}$C] CV-6504 from tissues and plasma. The concentration of CV-6504 and its metabolites in liver, kidney, tumour and plasma over a single 24 h period and six daily administrations is shown in Table I. Peak plasma levels of CV-6504 were observed at 0.25 h after administration. Free CV-6504 rapidly disappeared from the plasma and tissues and there was an accumulation of the sulphate and glucuronide, together with unknown metabolites. The concentration of unchanged CV-6504 and metabolites recovered from the tumour over the first 0.5 h of treatment was significantly lower than those found in the liver and similar to that found in kidney. However, by 24 h after a single administration or after six consecutive daily doses, the concentration of free CV-6504 in the tumour was significantly higher than the liver. The concentration of CV-6504 in the MAC16 tumour (3.3 μM) was equal to the concentration causing 50% inhibition of growth in tissue culture (3 μM), and, thus, sufficient to explain tumour regression. In both liver and tumour the concentration of CV-6504 glucuronides decreased with time, possibly owing to metabolism by β-glucuronidase. Measurement of enzyme activity in tissue homogenates showed similar activity to β-glucuronidase in MAC16 tumour and liver (10.8 and 10.3 μg phenolphthalein released from phenolphthalein glucuronide per mg protein in 30 min at pH 6.8). This confirms that the tumour has the ability to accumulate free CV-6504 by hydrolysis of the glucuronide conjugate.

The effect of CV-6504, the 1- and 4-glucuronide and the 1- and 4-sulphate metabolites on growth of the MAC13 cells *in vitro* is shown in Figure 1. While free CV-6504 effectively inhibited cell growth with an $IC_{50}$ value of 3 μM, none of the metabolites were effective growth inhibitors at concentrations up to 100 μM. The effect of the four metabolites and free CV-6504 on the growth of the MAC13 tumour *in vivo* is shown in Figure 2 (a and b). When administered daily at a dose of 50 mg kg$^{-1}$, the anti-tumour activity of the glucuronide and sulphate conjugates was similar to that obtained with free CV-6504 administered orally at 5 mg kg$^{-1}$ day$^{-1}$. The anti-tumour activity of the 4-glucuronide and 1-sulphate was slightly reduced in comparison with the 1-glucuronide and 4-sulphate, although this was not significant. This suggests that the MAC13 tumour may also be capable of enzymatic deconjugation of the glucuronide and sulphate metabolites. Broken cell preparations of the MAC13 tumour were capable of liberating 18 μg phenolphthalein per 30 min per mg protein from 0.4 mM phenolphthalein glucuronide and 30 nmol sulphate per 30 min per mg protein from 1.78 mM *p*-nitrocatechol sulphate at pH 5.0. Thus anti-tumour activity might be expected to be higher in those tumours expressing glucuronidase and sulphatase.

The effect of CV-6504 and the 1-sulphate and 4-glucuronide metabolites on the metabolism of AA in MAC13 tumours *ex vivo* is shown in Figure 3 (a and b). There was rapid metabolism of AA with fairly equal distribution along the pathways leading to the formation of 5-, 11-, 12- and 15-hydroxyeicosatetraenoic acids (HETEs). After incubation with 10 μM CV-6504 for 30 min, 5-, 11-, 12- and 15-HETE production, as well as the total unmetabolised AA, was significantly decreased. The percentage reduction in formation of 12- and 15-HETE exceeded that of 5-HETE. At a concentration of 100 μM, CV-6504 1-sulphate significantly reduced production of 12- and 15-HETE after 30 min, 11-HETE after 1 h and 5-HETE after 2 h. Incubation with

**Figure 2** The effect of CV-6504 (5 mg kg$^{-1}$ day$^{-1}$; ×) and (**a**) CV-6504-1-sulphate (●) and 4-sulphate (○) and (**b**) CV-6504-1-glucuronide (●) and 4-glucuronide (○) all at 50 mg kg$^{-1}$ day$^{-1}$ on the growth of the MAC13 tumour *in vivo*. Differences from control (□) values are indicated as a, $P<0.05$ and b, $P<0.01$, while c, $P<0.05$ from the group treated with CV-6504.

**Figure 3** The effect of CV-6504 (10 $\mu$M, 30 min; ■) and CV-6504-1-sulphate (**a**) (100 $\mu$M) or CV-6504-4-glucuronide (**b**) (100 $\mu$M) on the metabolism of AA to HETEs in the MAC13 tumour. Cells were untreated (▨) or treated with the metabolites for 30 min (▩), 1 h (□) or 2 h (▒). The experiment was repeated four times. Differences from controls are indicated as a, $P<0.01$ and b, $P<0.05$.

100 $\mu$M CV-6504 4-glucuronide also significantly reduced 5-, 11- and 12-HETE production by 30 min and 15-HETE production by 1 h.

### Discussion

We have previously shown CV-6504 to exert marked anti-tumour activity in the murine tumour models, MAC16 and MAC13, passaged in NMRI mice (Hussey *et al.*, 1996). CV-6504 is known to be rapidly removed from the circulation and converted into the glucuronide and sulphate conjugates, which are not effective lipoxygenase inhibitors and might not be expected to exert anti-tumour activity. It was, therefore, important to determine the rate and extent of formation of these metabolites in the murine model. After oral administration of [$^{14}$C] CV-6504, both free drug and metabolites were accumulated within the MAC16 tumour 2 h after administration. In comparison with liver, there was a significantly increased level of unmetabolised CV-6504 recovered per gram of tumour, which was at a concentration sufficient to account for the growth inhibition observed. At 24 h after oral dosing and after consecutive daily dosing, the relative concentration of unmetabolised to metabolised drug was increased, which was evident in the reduced percentage of glucuronide metabolites recovered. The glucuronide and sulphate conjugates of CV-6504 can be metabolised to free drug by the action of $\beta$-glucuronidase and sulphatase. Although the glucuronide and sulphate conjugates were ineffective in inhibiting the growth of the MAC13 tumour *in vitro*, they were as effective as CV-6504 *in vivo*, when used at a concentration five times higher. Both the MAC16 and MAC13 tumours possessed $\beta$-glucuronidase and sulphatase at levels similar to that found in the liver. This suggests a role for these enzymes in the anti-tumour action of CV-6504. A similar activation was observed for aniline mustard, which was metabolised to a glucuronide conjugate and was most active in tumours possessing $\beta$-glucuronidase activity (Connors and Whisson, 1966). Both $\beta$-glucuronidase and sulphatase are more active at lower pH values, which may be attained in solid tumours. A low pH would also facilitate uptake of the glucuronide conjugate into the cell by suppressing the ionisation of the carboxyl group.

Arachidonic acid can be oxygenated by a family of non-haem iron-containing dioxygenases – the lipoxygenases. The major mammalian enzymes are the 5-, 12- and 15-lipoxygenases, which form the corresponding 5-, 12- and 15-HETE. Both 12- and 15-HETE have been implicated in the stimulation of DNA synthesis and cell growth in fetal bovine aortic endothelial cells (Setty *et al.*, 1987), which is mediated by inhibition of diacyl glycerol kinase and the concomitant accumulation of cellular diacylglycerol. 12(S)-HETE has also been shown to promote wound healing of injured microvascular endothelial cells by increasing DNA synthesis more than 4-fold (Tang *et al.*, 1995). In neonatal rat lens epithelial cells it has been suggested that 12(S)-HETE may mediate EGF/insulin-stimulated DNA synthesis by regulating protooncogene expression (Lysz *et al.*, 1994), and that it may augment the invasiveness of rat prostatic tumour cells through selective activation of protein kinase $C_\alpha$ (Liu *et al.*, 1994*b*). Thus, inhibition of 12- and/or 15-HETE formation may be expected to inhibit tumour cell growth and metastasis.

In the present study, CV-6504 has been shown to inhibit the production of 5-, 12- and 15-HETE in MAC13 tumour cells *ex vivo*. Both the 12- and 15-lipoxygenase pathways are inhibited, despite the targeting of this compound to 5-lipoxygenase (Ohkawa *et al.*, 1991*a*), and the inhibitory effect on 12- and 15-lipoxygenase exceeded that of 5-lipoxygenase as measured by HETE formation. A similar effect has been observed with the PUFA eicosapentaenoic acid (EPA), which also exerts a marked antiproliferative effect on the chemounresponsive MAC16 tumour (Hudson *et al.*, 1993). This agent has also been shown to exert an anti-tumour effect against human breast cancer cells xenotransplanted into nude mice by reducing the tumour concentration of 12- and 15-HETE, while the level of 5-HETE was unaffected (Rose *et al.*, 1995). Both CV-6504 1-sulphate and 4-glucuronide inhibited formation of 5-, 12- and 15-HETE formation in MAC13 tumour cells *ex vivo* in a time-dependent manner, suggesting conversion of these metabolites to free CV-6504. Inhibition of 12- and 15-HETE production occurred before inhibition of 5-HETE production. Thus, suppression of 12- and/or 15-lipoxygenase pathways may be most important for inhibition of tumour growth.

These results suggest that CV-6504 may inhibit tumour growth as a result of the ability to inhibit 12- and/or 15-HETE production. Human tumours with a dependence on the 12- and 15-lipoxygenase pathways for growth may also be sensitive to this agent.

**Acknowledgements**
This work has been supported by a grant from Takeda Chemical Industries, Japan. We thank Mr M Wynter for the tumour transplantations.

### References

BANDYOPADHYAY GK, IMAGAWA W, WALLACE DR AND NANDI S. (1988). Proliferative effects of insulin and epidermal growth factor on mouse mammary epithelial cells in primary culture. Enhancement by hydroxyeicosatetraenoic acids and synergism with prostaglandin $E_2$. *J. Biol. Chem.*, **263**, 7567–7573.

BUCKMAN DK, HUBBARD NE AND ERICKSON KL. (1991). Eicosanoids and linoleate-enhanced growth of mouse mammary tumor cells. *Prostaglandins Leukotrienes Essential Fatty Acids*, **44**, 177–184.

CONNORS TA AND WHISSON ME. (1996). Cure of mice bearing advanced plasma cell tumours with aniline mustard: the relationship between glucuronidase activity and tumour sensitivity. *Nature*, **210**, 866–867.

GLASGOW WC AND ELING TE. (1990). Epidermal growth factor stimulates linoleic acid metabolism in BALB/C 3T3 fibroblasts. *Mol. Pharmacol.*, **38**, 503–510.

HUDSON EA, BECK SA AND TISDALE MJ. (1993). Kinetics of the inhibition of tumour growth in mice by eicosapentaenoic acid-reversal by linoleic acid. *Biochem. Pharmacol.*, **45**, 2189–2194.

HUSSEY HJ, BIBBY MC AND TISDALE MJ. (1996). Novel antitumour activity of 2, 3, 5-trimethyl-6-(3-pyridylmethyl)-1,4-benzoquinone against established murine adenocarcinomas. *Br. J. Cancer*, **73**, 1187–1192.

LEE P-PH AND IP MM. (1992). Regulation of proliferation of rat mammary tumor cells by inhibitors of cyclooxygenase and lipoxygenase. *Prostaglandins Leukotrienes Essential Fatty Acids*, **45**, 21–31.

LIU B, MARNETT LJ, CHAUDHARY A, JI C, BLAIR IA, JOHNSON CR, DIGLIO CA AND HONN KV. (1994*a*). Biosynthesis of 12(S)hydroxyeicosatetraenoic acid by B16 amelanotic melanoma cells is a determinant of their metastatic potential. *Lab. Invest.*, **70**, 314–323.

LIU B, MAHER RJ, HANNUN YA, PORTER AT AND HONN KV. (1994*b*). 12(S)-HETE. Enhancement of prostate tumor cell invasion: selective role of PKC$\alpha$. *J. Natl Cancer Inst.*, **86**, 1145–1151.

LYSZ TW, ARORA JK, LIN C AND ZELENKA PS. (1994). 12(S)-Hydroxyeicosatetraenoic acid regulates DNA synthesis and protooncogene expression induced by epidermal growth factor and insulin in rat lens epithelium. *Cell Growth Differentiation*, **5**, 1069–1076.

OHKAWA S, TERAO S, TERASHITA Z, SHIBOUTA Y AND NISHIKAWA K. (1991*a*). Dual inhibitors of thromboxane $A_2$ synthase and 5-lipoxygenase with scavenging activity of active oxygen species. Synthesis of a novel series of (3-pyridylmethyl)benzoquinone derivatives. *J. Med. Chem.*, **34**, 267–276.

OHKAWA S, TERAO S, MURAKAMI M, MATSUMOTO T AND GOTO G. (1991*b*). Reduction of 2,3,5-trimethyl-6-(3-pyridylmethyl)-1,4-benzoquinone by PB-3c cells and biological activity of its hydroquinone. *Chem. Pharm. Bull.*, **39**, 917–921.

RAO GN, ALEXANDER RW AND RUNGE MS. (1995). Linoleic acid and its metabolites hydroperoxyoctadecadienoic acids stimulate cFos, c-June and c-Myc mRNA expression, mitogen-activated protein kinase activation, and growth in rat aortic smooth muscle cells. *J. Clin. Invest.*, **96**, 842–847.

ROSE DP, CONNOLLY JM, RAYBURN J AND COLEMAN M. (1995). Influence of diets containing eicosapentaenoic or docosahexaenoic acid on growth and metastasis of breast cancer cells in nude mice. *J. Natl Cancer Inst.*, **87**, 587–592.

SETTY BNY, GRAEBER JE AND STUART MJ. (1987). The mitogenic effect of 15- and 12-hydroxyeicosatetraenoic acid on endothelial cells may be mediated via diacylglycerol kinase inhibition. *J. Biol. Chem.*, **262**, 17613–17622.

SIMON A, NAJID A, CHULIA AJ, DELAGE C AND RIAGAUD M. (1992). Inhibition of lipoxygenase activity and HL60 leukemic cell proliferation by ursolic acid isolated from heather flowers (*Calluna vulgaris*). *Biochim. Biophys. Acta*, **1125**, 68–72.

TANG DG, RENAUD C, STOJAKOVIC S, DIGLIO CA, PORTER A AND HONN KV. (1995). 12(S)-HETE is a mitogenic factor for microvascular endothelial cells: Its potential role in angiogenesis. *Biochem. Biophys. Res. Commun.*, **211**, 462–466.